# Mitochondrial genome characteristics and phylogenetic analysis of *Ramaria longispora*

**DOI:** 10.1080/23802359.2026.2728263

**Published:** 2026-09-05

**Authors:** Yan Yu, Chang Liu

**Affiliations:** ^a^ Department of Science and Technology, Luzhou Vocational and Technical College, Luzhou, Sichuan, China; ^b^ School of General Education, Luzhou Vocational and Technical College, Luzhou, Sichuan, China

**Keywords:** Mitochondrial genome, *Ramaria*, intron, phylogenetic analysis, basidiomycota

## Abstract

This study, for the first time, assembled and annotated the complete mitochondrial genome of *R. longispora* using high-throughput sequencing technology. The genome is a circular molecule with a total length of 157,712 bp and a GC content of 31.55%. It encodes 71 genes, including 15 core protein-coding genes (PCGs), 25 transfer RNA (tRNA) genes, 2 ribosomal RNA (rRNA) genes, 5 free-stranding open reading frames (ORFs), and 24 intronic ORFs. Among these, most free-stranding ORFs have unknown functions but include a DNA polymerase gene, while the intronic ORFs primarily encode LAGLIDADG and GIY-YIG endonucleases. The mitochondrial genome contains 39 introns. Phylogenetic analyses based on 15 core PCGs using Bayesian inference (BI) and maximum likelihood (ML) methods revealed that this *R. longispora* is most closely related to *Ramaria flavescens* and *Ramaria ichnusensis*. This study provides foundational data for mitochondrial genome research in the *Ramaria* genus and offers important references for taxonomic and evolutionary studies of this group.

## 1. Introduction

The genus *Ramaria*, belonging to Basidiomycota, Agaricomycetes, and Gomphales, is a widely distributed group of coral-like fungi with significant ecological functions and economic value (Toledo et al. 2016; Ozen et al. 2019; Schön et al. 2023). Most species in this genus are ectomycorrhizal, forming symbiotic relationships with trees such as Pinaceae and Fagaceae, playing a crucial role in material cycling and community construction within forest ecosystems (Nouhra et al. 2005; Lupatini et al. 2008). Additionally, some *Ramaria* species possess edible or medicinal value, attracting researchers’ attention (Li et al. 2025; Liu et al. 2025; Malacara-Marquez et al. 2026). However, the complex morphological characteristics and ambiguous interspecific boundaries within this genus pose limitations for traditional taxonomic studies, necessitating supplementation with molecular phylogenetic data.

The mitochondrial genome has become an important molecular marker in fungal phylogenetics, population genetics, and evolutionary biology research due to its conserved structure, moderate evolutionary rate, and maternal inheritance characteristics (Chen et al. 2026; Pérez et al. 2026). In recent years, with the development of high-throughput sequencing technologies, fungal mitochondrial genomics has made significant progress, providing new perspectives for resolving the phylogenetic relationships among fungal taxa (Liang et al. 2026; Viljoen et al. 2026). For example, Li et al. (Li et al. 2022) first reported the mitochondrial genomes of two *Ramaria* species, revealing the evolutionary features of this group in terms of mitochondrial gene arrangement and intron dynamics. However, current data on the mitochondrial genomes of the *Ramaria* genus remain relatively limited, hindering a comprehensive understanding of their genomic diversity and evolutionary history (Wang et al. 2025).

This study is the first to sequence, assemble, and annotate the mitochondrial genome of *Ramaria longispora* Marr & D.E. Stuntz 1974, analyzing its structural characteristics, gene composition, and intron distribution. This research aims to enrich the mitochondrial genome data of the *Ramaria* genus and provide foundational information for taxonomic revisions and evolutionary biology studies of this genus.

## 2. Materials and methods

### 2.1. Sample collection and identification

**Figure 1.**
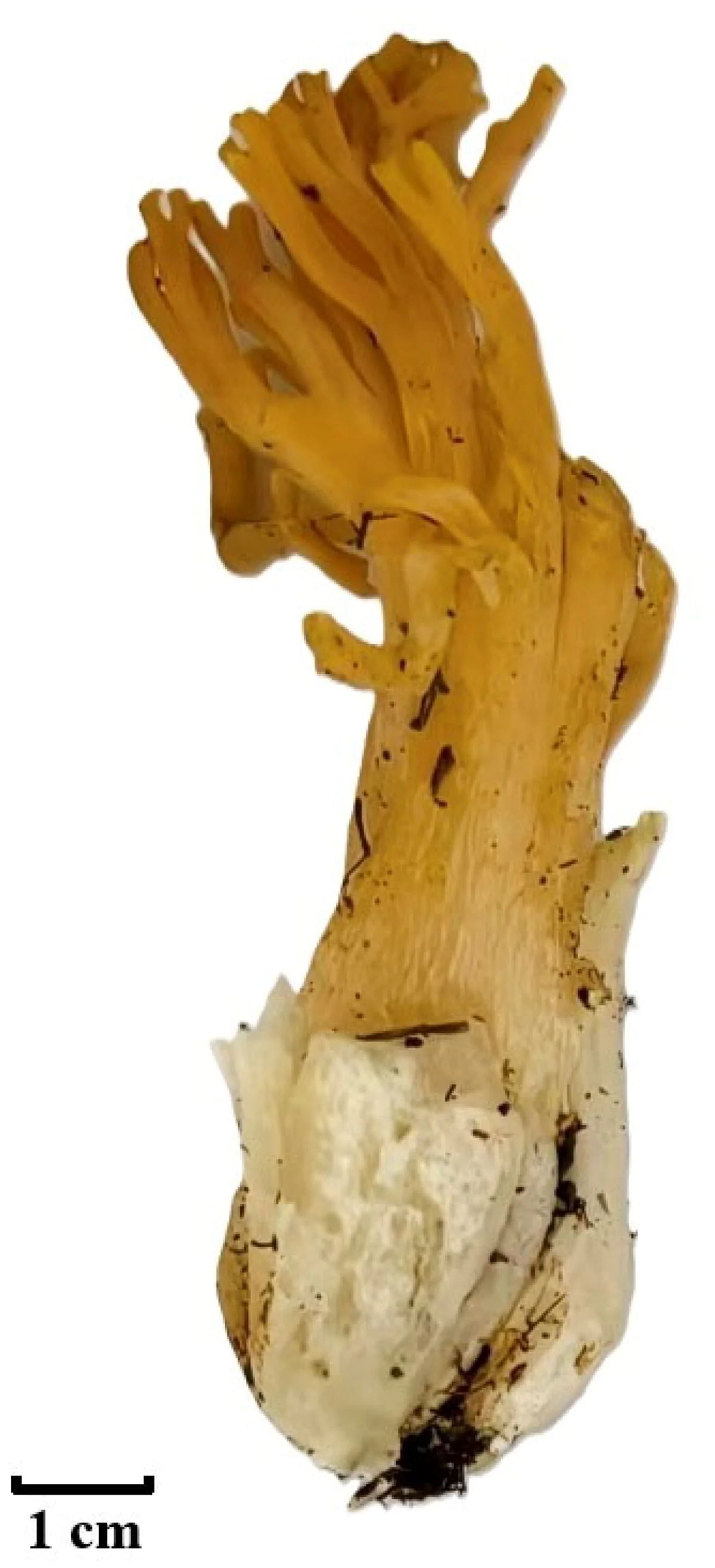
Basidiocarps of *R. longispora* in natural habitat, showing typical features: fleshy, centrally depressed pileus with a prominent umbo, decurrent lamellae, and a robust, tapering stipe with a distinct ring zone. Photo was taken by Yan Yu.

The fruiting bodies of *R. longispora* were collected in 2024 from a mixed coniferous and broad-leaved forest in Nanhua County (101.27 E, 25.24 N), Yunnan Province, China (Figure 1). After collection, the samples were immediately transported to the laboratory in an icebox. Preliminary identification was conducted based on the macroscopic morphological characteristics of the fruiting bodies, including branching patterns, color, and basal structures. For molecular identification, genomic DNA was extracted, and the ribosomal DNA internal transcribed spacer (ITS) sequence was amplified and sequenced (Li et al. 2025). Species affiliation was confirmed through NCBI BLAST comparison (99.35% similarity with *R. longispora* voucher MHHNU9241 PP493503). Voucher specimens are preserved at the Microbial Culture Collection Center of Luzhou Vocational and Technical College the accession number Rsp03 (contact person: Yu Yan; email: yuyan@lzy.edu.cn).

### 2.2. Mitochondrial genome sequencing, assembly, and annotation

The total DNA of the *R. longispora* fruiting body was extracted using the Fungal DNA Extraction Kit (Omega Bio-Tek, Norcross, GA, USA). The sequencing library was constructed using the NEBNext^®^ Ultra^™^ II DNA Library Prep Kit (New England Biolabs, Beijing, China) and subjected to paired-end sequencing (2 × 150 bp) on the Illumina HiSeq 2500 platform. Raw sequencing data underwent quality control using ngsShoRT (Chen et al. 2014) and AdapterRemoval v2 (Schubert et al. 2016) to remove low-quality sequences and adapter contamination. *De novo* assembly of the mitochondrial genome was performed using NOVOPlasty v4.3.3 (Dierckxsens et al. 2017) with a k-mer size set to 33. Preliminary annotation was conducted using the MFannot (Valach et al. 2014) and MITOS (Bernt et al. 2013) web servers, combined with NCBI ORF Finder (Coordinators 2017) to predict open reading frames (ORFs) longer than 100 amino acids. Functional annotation was performed *via* BLASTP alignment against the NCBI non-redundant protein database (Bleasby and Wootton 1990). tRNA genes were identified and validated using tRNAscan-SE v1.3.1 (Lowe and Chan 2016). The circular mitochondrial genome map and cis-splicing genes were generated using PMGmap (http://www.1kmpg.cn/pmgmap) (Zhang et al. 2024). Intron types were determined by alignment with the Rfam database and published literature (Slater and Birney 2005).

### 2.3. Phylogenetic analysis

To determine the phylogenetic position of *R. longispora*, 15 conserved mitochondrial core protein-coding genes (*atp6*, *atp8*, *atp9*, *cob*, *cox1*, *cox2*, *cox3*, *nad1*, *nad2*, *nad3*, *nad4*, *nad4L*, *nad5*, *nad6*, and *rps3*) were selected for phylogenetic reconstruction. Corresponding sequences from 10 other basidiomycetes were downloaded from the NCBI GenBank database (species list and accession numbers are shown in Figure 3), with *Tuber calosporum* used as the outgroup (Li et al. 2020). Each gene was individually aligned using MAFFT v7.037 (Katoh et al. 2019), and after removing intron regions, concatenation was performed using SequenceMatrix v1.7.8 (Vaidya et al. 2011). The optimal partitioning scheme and nucleotide substitution models were determined using PartitionFinder 2.1.1 (Lanfear et al. 2017). Bayesian Inference (BI) and Maximum Likelihood (ML) methods were employed to construct phylogenetic trees. BI analysis was conducted using MrBayes v3.2.6 (Ronquist et al. 2012). ML analysis was performed using RAxML v8.0.0 (Stamatakis 2014), with 10,000 rapid bootstrap replicates to assess node support.

## 3. Results

**Figure 2.**
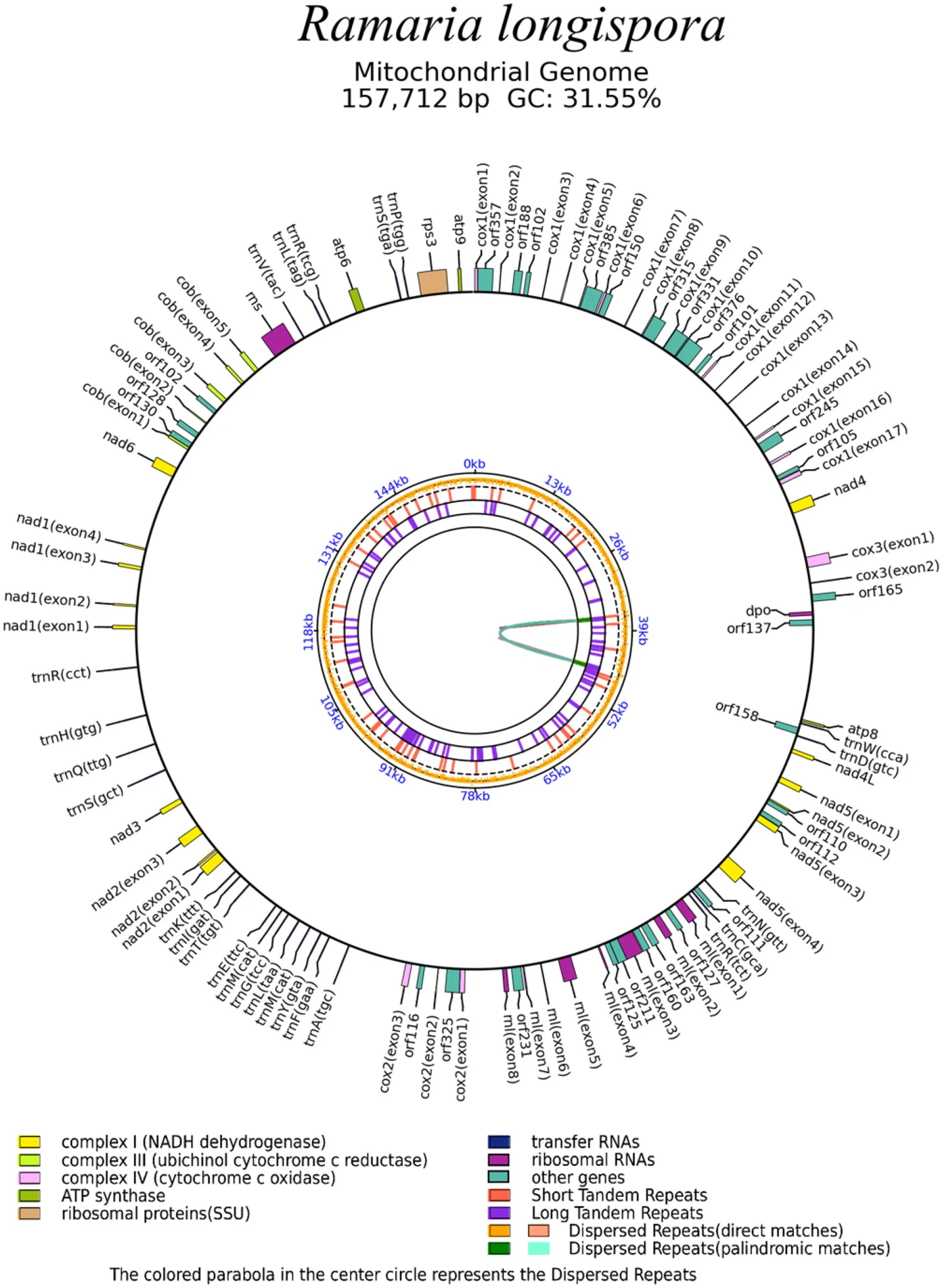
The circular mitochondrial genome map of *R. longispora* colored blocks outside each ring indicate that the genes are on the direct strand, while colored blocks within the ring indicate that the genes are located on the reverse strand. The colored parabola in the center circle represents the dispersed repeats.

The mitochondrial genome sequencing of *R. longispora* had an average coverage depth of 577.59× (Supplementary Figure 1). After assembly, a circular mitochondrial genome was obtained (Figure 2), with a total length of 157,712 bp and a GC content of 31.55%. The base composition exhibited asymmetry, with higher proportions of A (33.67%) and T (34.77%), and lower proportions of G (14.85%) and C (16.70%).

Genome annotation identified a total of 71 genes, including 15 core protein-coding genes (*atp6*, *atp8*, *atp9*, *cob*, *cox1*, *cox2*, *cox3*, *nad1*, *nad2*, *nad3*, *nad4*, *nad4L*, *nad5*, *nad6*, and *rps3*), 2 rRNA genes (*rnl* and *rns*), 25 tRNA genes, 5 free-stranding ORFs, and 24 intronic ORFs. Among the free-stranding ORFs, except for one encoding a DNA polymerase, the rest encoded proteins of unknown function. The intronic ORFs primarily encoded LAGLIDADG and GIY-YIG family endonucleases.

The mitochondrial genome contains a total of 39 introns, distributed across multiple protein-coding genes (Supplementary Figure 2). Based on their structural and conserved sequence characteristics, these introns can be classified into the following types: 17 Group IB, 6 Group I (derived), 4 Group IA, 2 Group IC, 2 Group ID, 1 Group II, and 7 introns of undetermined type.

**Figure 3.**
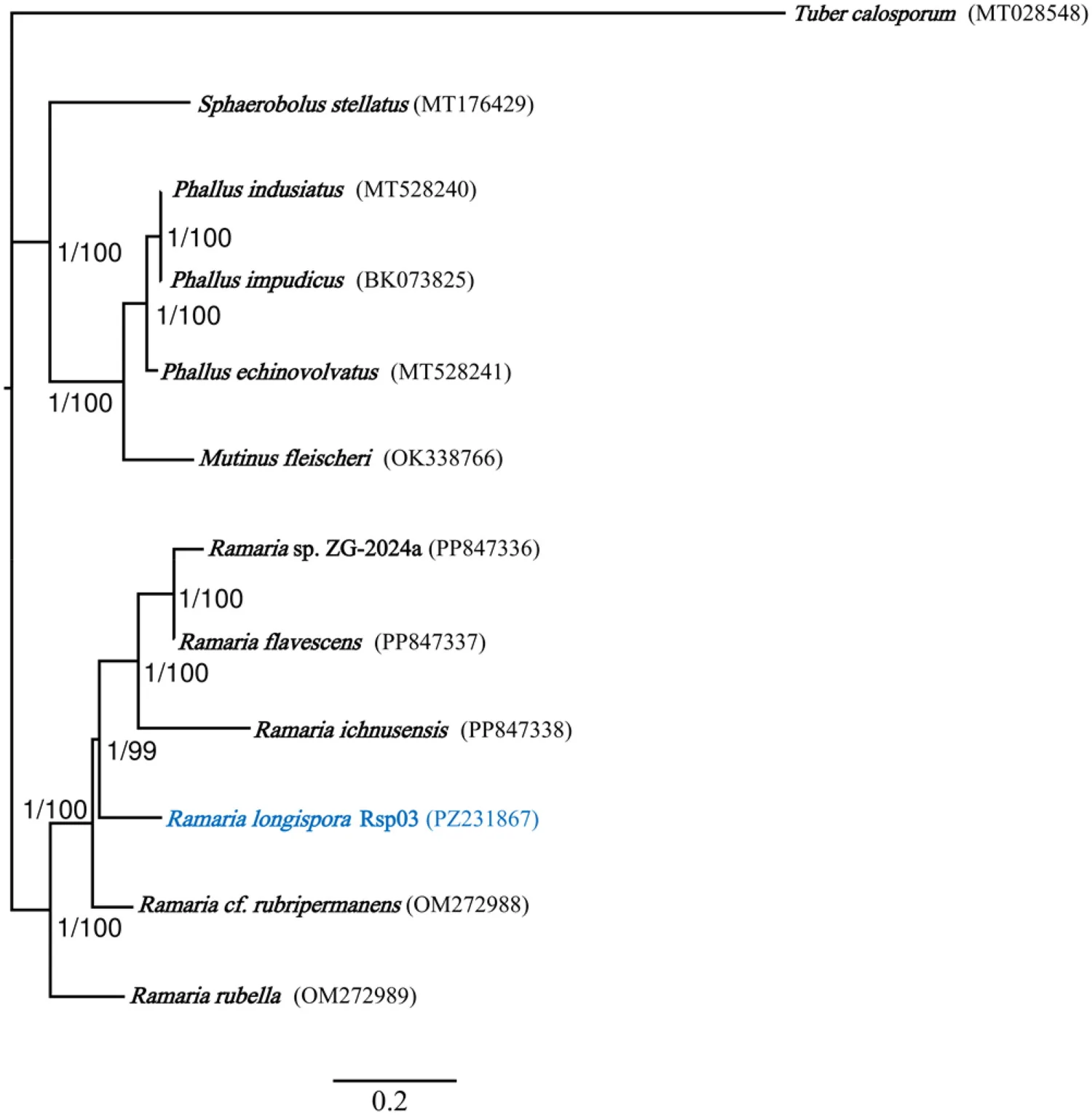
Bayesian Inference and maximum likelihood tree (a) generated using 15 concatenated mitochondrial protein-coding genes (*atp6*, *atp8*, *atp9, cob, cox1, cox2, cox3, nad1, nad2, nad3, nad4, nad4L, nad5, nad6,* and *rps3*) from *R. longispora* and 11 other fungal species. The support values are Bayesian posterior probabilities (BPPs) and bootstrap values (BSs). *Tuber calosporum* (MT028548) was used as the outgroup (Li et al. 2020). the accession number information of the sequence is as follows: *Sphaerobolus stellatus* (MT176429), *phallus indusiatus* (MT528240), *phallus impudicus* (BK073825), *phallus echinovolvatus* (MT528241), *mutinus fleischeri* (OK338766), *ramaria* sp. ZG-2024a (PP847336) (Wang et al. 2025), *ramaria flavescens* (PP847337) (Wang et al. 2025), *ramaria ichnusensis* (PP847338) (Wang et al. 2025), *R. longispora* Rsp03 (PZ231867) (Li et al. 2022), *ramaria cf. rubripermanens* (OM272988) (Li et al. 2022), and *ramaria rubella* (OM272989) (Li et al. 2022).

Phylogenetic analysis utilized a concatenated dataset of 15 core protein-coding genes. The topologies of the phylogenetic trees constructed by Bayesian inference (BI) and maximum likelihood (ML) methods were consistent, with high support values at all nodes (Figure 3). The results revealed that the *R. longispora* in this study clustered with *Ramaria flavescens* and *Ramaria ichnusensis* (Wang et al. 2025), indicating the closest phylogenetic relationship. This suggests that the species belongs to the genus *Ramaria* and shares a more recent common ancestor with the aforementioned two species.

## 4. Discussion and conclusion

This study reports for the first time the complete mitochondrial genome of *R. longispora*, revealing its genomic structure, gene composition, intron distribution characteristics, and reconstructing its phylogenetic relationships based on core protein-coding genes. The study found that the mitochondrial genome of *R. longispora* is significantly larger than that of most other Basidiomycetes (Wang et al. 2025), which is closely related to its intron-rich features (Li et al. 2021, 2022, 2023). *R. longispora* contains 38 introns, a number far exceeding those previously reported in *R. rubella* and *R. cf. rubripermanens* (Li et al. 2022), suggesting significant intron dynamics may exist within the genus *Ramaria*. As ‘selfish’ genetic elements, introns are widespread in fungal mitochondrial genomes, and their insertion and loss are important driving forces for the size and structural variation of fungal mitochondrial genomes (Wang et al. 2020; Ma et al. 2022). This study identified various types of Group I introns, as well as a Group II intron, reflecting the high diversity of intron composition in the mitochondrial genome of *R. longispora*

It is noteworthy that this study identified a DNA polymerase gene within the independent ORFs of *R. longispora*, which may be involved in the replication and repair processes of mitochondrial DNA (Li et al. 2019, 2020; Wang et al. 2020; Huang et al. 2021). Previously, DNA polymerase genes were not commonly found in fungal mitochondrial genomes, existing more frequently as nuclear genes (Li et al. 2018, 2019, 2024). Their presence in the mitochondrial genome of *R. longispora* might be attributed to horizontal gene transfer or gene migration events, and their functional and evolutionary significance warrants further investigation.

## Supplementary Material

Supplementary_figure-Clean.docx

## Data Availability

The genome sequence data that support the findings of this study are openly available in the NCBI GenBank at https://www.ncbi.nlm.nih.gov/under accession no. PZ231867. The associated BioProject, SRA, and BioSample numbers are PRJNA1446173, SRR37894749 and SAMN56813425, respectively.
