# Supplementary material for: Mitochondrial genome characteristics and phylogenetic analysis of Ramaria longispora
Source: Mitochondrial DNA B Resour. 2026 Sep 5;11(10):1128–33. doi: 10.1080/23802359.2026.2728263 (PMC13552289; doi:10.1080/23802359.2026.2728263)
Supplement: Supplementary file 1 [file TMDN_A_2728263_SM3570.docx]

**Supplementary Figure S1** Sequencing depth and coverage map of *Ramaria longispora* mitochondrial genome.

**Supplementary Figure S2** Cis-splicing genes of *Ramaria longispora* mitochondrial genome.
